# Distribution of sika deer (*Cervus nippon*) and the bioclimatic impact on their habitats in South Korea

**DOI:** 10.1038/s41598-023-45845-2

**Published:** 2023-11-03

**Authors:** Thakur Dhakal, Tae-Su Kim, Seong-Hyeon Kim, Shraddha Tiwari, Jun-Young Kim, Gab-Sue Jang, Do-Hun Lee

**Affiliations:** 1 Department of Life Sciences, Yeungnam University, Gyeongsan, 38541 Republic of Korea; 2 College of Veterinary Medicine, Kangwon National University, Chuncheon, 24341 Republic of Korea; 3 National Institute of Ecology, Seocheon, 33657 Republic of Korea

**Keywords:** Ecology, Climate sciences

## Abstract

Invasive species and climate change are primary factors influencing biodiversity, and examining the behavior of invasive species is essential for effective conservation management. Here, we report the global distribution of the sika deer (*Cervus nippon*) based on locations reported in published literature (Google Scholar), the Global Biodiversity Information Facility (GBIF) database, and the International Union for Conservation of Nature report. We used the maximum entropy (Maxent) model to examine the impact of climate change on sika deer habitats in South Korea based on GBIF occurrence data and WorldClim bioclimatic variables. Habitat suitability analysis was performed using the Maxent model under Representative Concentration Pathways (RCPs) 4.5 and 8.5 (for predicted climatic conditions in both 2050 and 2070) to project the effects of different climate change scenarios on South Korean sika deer habitats. We identified that the sika deer is distributed in 39 countries worldwide. Due to climate change effects, South Korean sika deer habitats will decline by approximately 24.98% and 20.63% (under RCP 4.5) and by 50.51% and 57.35% (under RCP 8.5) by 2050 and 2070, respectively. Our findings shed light on sika deer ecology and provide reference data for future conservation management strategies and policy design.

## Introduction

Sika deer (*Cervus nippon*; Cervidae: Cervinae: *Cervus*) are native to Japan, Taiwan, and eastern Asia^1,2^, and the species has been introduced to several countries worldwide at different times for various purposes^3^. The International Union for Conservation of Nature (IUCN; www.iucnredlist.org) recognizes the sika deer as an endangered species in specific regions and countries^1,2^. However, in other regions, sika deer—similar to other invasive species—cause dramatic biodiversity loss, negatively affect native species, and pose a challenge to conservation management strategies^4,5^. Therefore, appropriate monitoring and inventory programs and the collection of quality data regarding the distribution of this species are needed for effective conservation management^6^.

Invasive species and climate change are considered major threats to native biodiversity, and both are studied extensively^7–9^. Therefore, habitat suitability modeling and landscape-oriented management strategies have been developed to support the management of invasive species^10,11^. For example, factors such as snow depth, human disturbance, vegetation cover, and topological factors are known to affect sika deer habitats and have been studied extensively^1,6,12,13^. However, there is limited information regarding the impact of the bioclimatic variables of climate change on sika deer habitats.

In South Korea, the native deer are extinct^14^ and the invasive sika deer, introduced from Japan and Taiwan, is one out of approximately 2160 (334 plants and 1826 animals) alien species^15,16^. Alien species in South Korea have more than doubled over the last decade^15,17^. Deer populations are also increasing in Korea, but the exact number is undefined^18^. The increase in biodiversity due to invasive species increases the conservation value of local ecosystems^19^. Member organizations of the IUCN and ecologists are actively working on identifying invasive species diversity.

The Ministry of Environment, South Korea has shaped a strategy to predict the current distribution and potential spread of invasive species for sustainable biodiversity and wildlife management^17^. The current study therefore aimed to investigate the present worldwide distribution of sika deer and how the distribution in South Korea may interact in future under the influence of climate change using the maximum entropy (Maxent) habitat suitability model^20^ and bioclimatic variables^21^. This research is expected to provide valuable insights into the current and potential future distribution of sika deer worldwide and the impact of climate change on their habitats, with a particular focus on South Korea's unique ecological context coupling with bioclimatic variables and the importance of effective conservation management.

## Materials and methods

### Distribution data

Distribution data for the sika deer were collected through two approaches: from the survey areas or sample collection sites of previous studies (found on Google Scholar) and existing occurrence data (obtained from the GBIF database). These data were compared with the information in the IUCN invasive species database^22^. Google Scholar is the most comprehensive repository of this literature and has higher performance than any other literature databases^23,24^. The GBIF database is an open international database that contains global biodiversity information^25^. A total of 977 studies containing the phrase ‘sika deer’ in the title were obtained from Google Scholar using Harzing’s Publish or Perish tool^26^. The occurrence data of sika deer were mined from the GBIF portal^25^ using the keyword ‘*Cervus nippon*’ on June 17, 2022; the ‘occ_data’ function in the R package *rgbif* (version 3.7.2.5) was used in the RStudio^27^, resulting in 8385 records of species presence data.

### Bioclimatic variables

A dataset containing bioclimatic variables for both current climate conditions (baseline) and projected climate scenarios was constructed to assess the impact of climate change^28–31^. In this study, 19 historical bioclimatic factors (bio1, bio2, bio3, bio4, bio5, bio6, bio7, bio8, bio9, bio10, bio12, bio13, bio14, bio15, bio16, bio17, bio18, and bio19) (Table 1) were mined from the WorldClim database^21^. These factors have been most commonly used over a long time frame (1970–2000, considered current data), and they were extracted from the database using the ‘getData’ function in the *raster* package in the RStudio environment^32,33^. Similarly, future bioclimatic variables for 2050 and 2070 that projected based on Representative Concentration Pathways (RCP) 4.5 and 8.5^34^ were downloaded from the Coupled Model Intercomparison Project Phase 5 (CMIP5)^35^ application programming interface in RStudio^32,33^. The RCP scenarios used in this study are predetermined pathways for levels of greenhouse gas and aerosol concentrations, along with changes in land use, which align with a collection of general climate results employed by the climate modeling community^36–38^. The general and interaction effects can be examined without multicollinearity test^39^ and the current study examined all 19 bioclimatic factors.

**Table 1:** Bioclimatic variables from the WorldClim database and their codes and units of measurement.

Bioclimatic variable	Code	Unit
Annual Mean Temperature	bio1	°C
Mean Diurnal Range (Mean of monthly (max temp—min temp))	bio2	°C
Isothermality (BIO2/BIO7) (× 100)	bio3	%
Temperature Seasonality (standard deviation × 100)	bio4	°C
Max Temperature of Warmest Month	bio5	°C
Min Temperature of Coldest Month	bio6	°C
Temperature Annual Range (BIO5—BIO6)	bio7	°C
Mean Temperature of Wettest Quarter	bio8	°C
Mean Temperature of Driest Quarter	bio9	°C
Mean Temperature of Warmest Quarter	bio10	°C
Mean Temperature of Coldest Quarter	bio11	°C
Annual Precipitation	bio12	mm
Precipitation of Wettest Month	bio13	mm
Precipitation of Driest Month	bio14	mm
Precipitation Seasonality (Coefficient of Variation)	bio15	%
Precipitation of Wettest Quarter	bio16	mm
Precipitation of Driest Quarter	bio17	mm
Precipitation of Warmest Quarter	bio18	mm
Precipitation of Coldest Quarter	bio19	mm

### The Maxent model

The Maxent model is an extensively used density estimation technique where the probability distribution (π) for a set of data (*X*) is estimated based on the species presence data and spatial variables in the study area^40,41^. The presence data in set *X* is set to 1 and 0 for presence and absence, respectively, the response variable is *Y*, and the distribution π(*X*) is the conditional probability $P(X|Y\, = \,{1})$^20^. The Maxent method follows the Bayesian rule (Eq. 1):

1 $$P\left( {Y = 1{|}X} \right) = \frac{{P\left( {X{|}Y = 1} \right)P\left( {Y = 1} \right)}}{P\left( X \right)} = \pi \left( X \right)P\left( {Y = 1} \right)\left| X \right|$$

In the current study, we used the Maxent model suggested by Phillips et al.^20,42^ and implemented it in the *dismo* package in R^33^ to analyze the impact of climatic changes on the habitat of the sika deer. The geographical range of South Korea was used as a survey region to illustrate the impact of climatic changes. To fit the occurrence data of sika deer to the Maxent model, we filtered the presence data mined from the GBIF database within the South Korean region but obtained only seven coordinates with confirmed species identification. Model performance decreases rapidly with a sample size of < 20^43^ or < 15^44^ and is dramatically poorer for sample sizes of < 5^45^. Therefore, we used all species occurrence data points (6277 locations with species identification and the associated geo-coordinates) with the assumptions of spatial independence to train and test the Maxent model and projected our results onto the survey area. The data were grouped randomly as follows: 75% for model training (4707 data points; 5000 replications) and 25% for model testing (1570 points). Maxent is computed with presence data against the pseudo-absence data (or background data), whose sample size needs to be more than 10,000 data points for larger datasets^46^. Therefore, we generated 10,000 random locations as background data to evaluate the Maxent model with the testing dataset and used current bioclimatic variables as environmental predictors.

Model performance was evaluated using the area under the curve (AUC)^20^, Cohen’s Kappa^47^, and true skill statistic (TSS)^48^ in order to estimate the probable habitats of the sika deer based on climatic attributes^42,49^. The AUC values range between 0 and 1, and the results were interpreted as follows: 0.9–1.0, best agreement; 0.8–0.9, good agreement; 0.7–0.8, fair agreement; 0.7, poor agreement^50^. Cohen’s Kappa ranges between − 1 and + 1 (0.80–1.0, best agreement; 0.60–0.80, substantial agreement; 0.40–0.60, moderate agreement; 0.20–0.40, fair agreement; 0.01–0.20, slight to no agreement; ≤ 0, no agreement)^51^. The TSS values range from − 1 to + 1, with + 1 indicating perfect agreement and ≤ 0 indicating a performance no better than random^48^.

## Results

### Sika deer distribution

**Figure 1:**
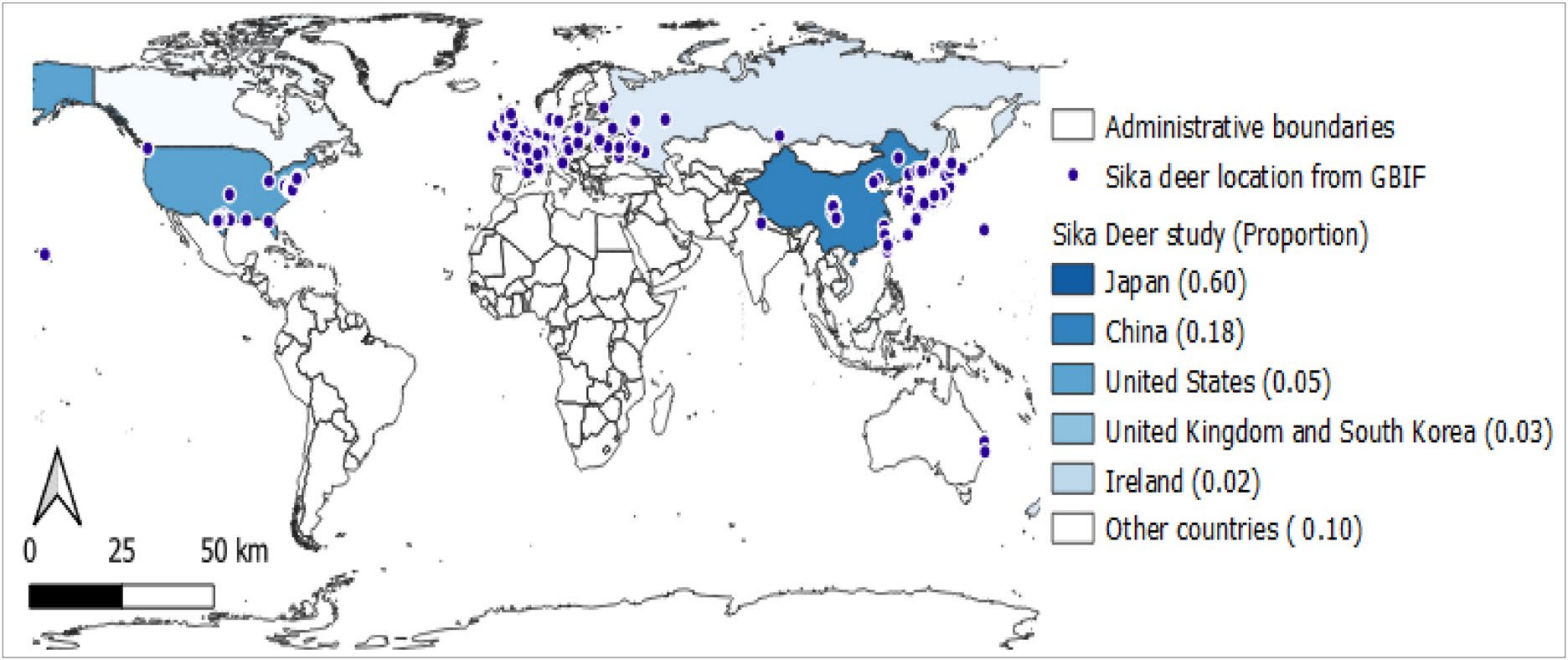
Distribution of sika deer based on Global Biodiversity Information Facility (GBIF) occurrence data and sample collection sites from 977 Google Scholar indexed studies. (Map generated in QGIS Desktop 3. 24.1.; Base map obtained from CC BY 4.0 licensed GADM database https://gadm.org/index.html).

We examined the global distribution of the sika deer using two approaches: experimental data sourced from 977 published studies (Google Scholar) and 8385 species occurrence data points obtained from the GBIF database. The combined data indicated that the sika deer is distributed in 34 countries (Australia, Austria, Belarus, Belgium, Canada, China, Croatia, Czech Republic, Denmark, Estonia, France, Germany, India, Ireland, Japan, Kenya, Lithuania, Luxembourg, Malaysia, Mauritius, Netherlands, New Zealand, North Korea, Poland, Russia, South Korea, Switzerland, Taiwan, Tanzania, Thailand, Ukraine, United Kingdom, United States, and Vietnam) (Fig. 1). Of these, 25 countries were represented in previous literature, whereas 29 were represented in the GBIF database. Data pertaining to sika deer occurrences in Australia, Belarus, Croatia, Estonia, India, Kenya, and Tanzania were absent in the literature search, and sampling locations in Lithuania, Luxembourg, Malaysia, Mauritius, and North Korea were absent in the GBIF database. We compared these countries with those listed in the IUCN database, which records sika deer distributions in 24 countries and lists China, Japan, Korea, Russia, Taiwan, and Vietnam as the native range of this species (Table 2). Finally, we concluded that the sika deer is distributed in 39 countries worldwide.

**Table 2:** Sika deer occurrence in different countries worldwide, as recorded in different databases: International Union for Conservation of Nature (IUCN), Global Biodiversity Information Facility (GBIF), and Google Scholar literature search.

Countries with sika deer occurrence (present survey)	IUCN database	GBIF database	Literature search
Armenia, Australia, Austria, Azerbaijan, Belarus, Belgium, Canada, China, Croatia, Czech Republic, Denmark, Estonia, Finland, France, Germany, India, Ireland, Japan, Kenya, Lithuania, Luxembourg, Madagascar, Malaysia, Mauritius, Netherlands, New Zealand, North Korea, Philippines, Poland, Russia, South Korea, Switzerland, Taiwan, Tanzania, Thailand, Ukraine, United Kingdom, United States, Vietnam	Armenia, Austria, Azerbaijan, China, Czech Republic, Denmark, Finland, France, Germany, Ireland, Japan, North Korea, South Korea, Lithuania, Madagascar, New Zealand, Philippines, Poland, Russia, Taiwan, Ukraine, United Kingdom, United States, Vietnam	Australia, Austria, Belarus, Belgium, Canada, China, Croatia, Czech Republic, Denmark, Estonia, France, Germany, India, Ireland, Japan, Kenya, Netherlands, New Zealand, Poland, Russia, South Korea, Switzerland, Taiwan, Tanzania, Thailand, Ukraine, United Kingdom, United States, Vietnam	Austria, Belgium, Canada, China, Czech Republic, Denmark, France, Germany, Ireland, Japan, Lithuania, Luxembourg, Malaysia, Mauritius, Netherlands, New Zealand, North Korea, Poland, Russia, South Korea, Switzerland, Taiwan, United Kingdom, United states, Vietnam

**Figure 2:**
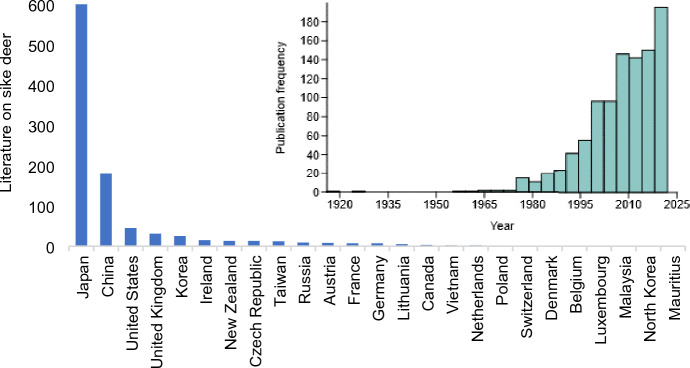
Studies on Sika deer obtained from Google Scholar database between 1918 to 2022/09/14 (Keyword ‘Sika Deer’ in the title and defined geographic location of examined Sika deer; inset histogram shows the publication frequencies).

Of the 977 studies on sika deer obtained from Google Scholar, most were related to sika deer in Japan (i.e., their native range; 601 studies, 59.92%), followed by China (181 studies, 18.05%), the United States (46 studies, 4.59%), the United Kingdom (32 studies, 3.19%), South Korea (26 studies, 2.59%), and Ireland (16 studies, 1.60%) (Figs. 1 and 2). Recent years have seen the publication of a number of studies on sika deer management, genetics, and ecology, and the publication frequency has been increasing since early in the twentieth century (Fig. 2).

### Impact of climatic factors on the distribution of sika deer in South Korea

The Maxent model was fitted based on the 19 bioclimatic variables and global distribution data for the sika deer (6277 data points). The model with maximum AUC (0.81), TSS (0.61), and Kappa (0.67) values was used to predict a potential map of suitable habitats for the sika deer based on current and future (2050 and 2070) climatic conditions at the moderate and most extreme scenarios (RCP 4.5 and 8.5, respectively). Among the 19 bioclimatic variables used in our analysis, bio14 had the highest (55.5%) relative contribution to the Maxent model (Table 3), followed by bio11 (16.7%), bio1 (7.4%), and bio3 (7.2%). When presence data were randomly permuted to the Maxent model, bio3 (43.6%) had the highest permutation importance, followed by bio1 (162%), bio12 (7.4%), and bio7 (7.2%). Overall, bio16 was the lowest contributor to sika deer habitat distribution in the Maxent model (for both percent contribution and permutation importance; Table 3).

**Table 3:** Percent contribution and permutation importance of each bioclimatic variable.

Variable	Percent contribution	Permutation importance	Variable	Percent contribution	Permutation importance
bio14	55.40	1.10	bio12	0.30	7.40
bio11	16.70	4.30	bio9	0.20	0.90
bio1	7.40	16.20	bio10	0.20	0.50
bio3	7.20	43.60	bio19	0.20	0.70
bio7	4.10	7.20	bio8	0.10	1.00
bio6	3.70	2.00	bio17	0.00	4.80
bio2	1.70	0.20	bio15	0.00	0.10
bio5	1.00	6.00	bio13	0.00	1.30
bio18	1.00	2.00	bio16	0.00	0.00
bio4	0.80	0.60			

**Figure 3:**
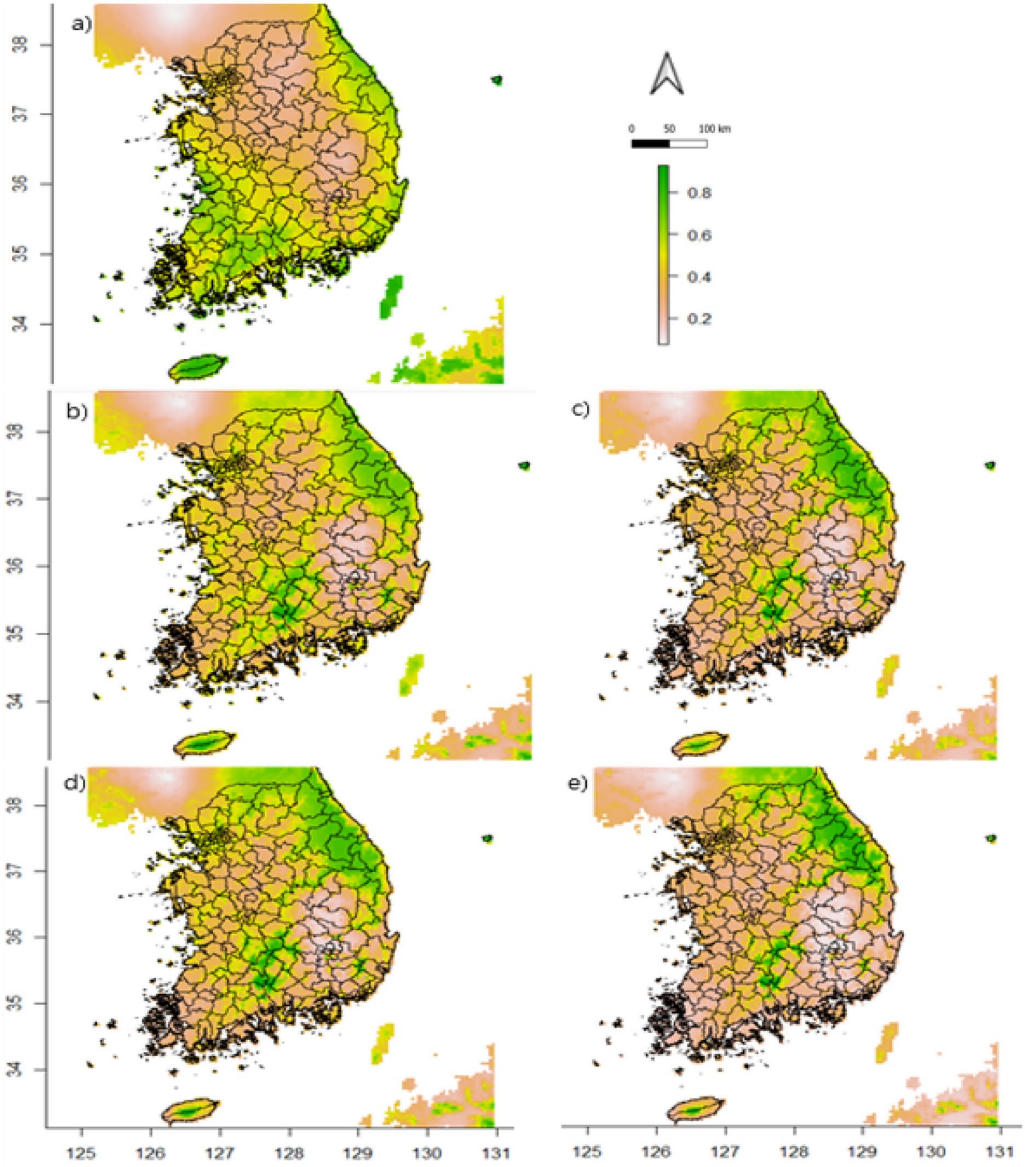
Potential distribution of sika deer habitats in South Korea mapped using Maxent models under climatic conditions: (**a**) current (*1970–2000)*, (**b**) Representative Concentration Pathway (RCP) 4.5 for 2050, (**c**) RCP 4.5 for 2070, (**d**) RCP 8.5 for 2050, and (**e**) RCP 8.5 for 2070 scenarios. (Color bar represents the entropy values; Maps generated in QGIS Desktop 3. 24.1; Base map obtained from CC BY 4.0 licensed GADM: https://gadm.org/index.html and WorldClim: https://www.worldclim.org/about.html, database).

The predicted model, along with current and future bioclimatic variables, was used to calculate the number of grids with entropy values greater than the Maxent threshold (0.65) within the Korean distribution range of this species. The suitable habitat range of the sika deer decreased in all climatic scenarios (Fig. 3). Our results indicated that by 2050 and 2070, the habitat range of the sika deer in South Korea will respectively decrease by 24.98% and 20.63% (under the RCP 4.5 scenario) and by 50.51% and 57.35% (under the RCP 8.5 scenario) compared with the current distribution range.

## Discussion

Invasive species management poses a significant challenge for current society, although its implementation can be improved by integrating management priorities^52^. The sika deer is an invasive species that has gone extinct in some countries and is overabundant in Japan^2,22^. Therefore, characterizing the distribution of this species and identifying its hotspots are essential for conservation biology^53^. We identified some discrepancies in the IUCN and GBIF databases for sika deer distribution and searched the existing literature to identify countries in which the sika deer has been sampled and researched. Taken together, our analysis of data from the three databases revealed that the sika deer is distributed in 39 countries (Table 2). In addition, the sampling locations recorded in previous studies and in the GBIF database indicate that the sika deer is predominantly distributed in the northern hemisphere (Fig. 1).

Several studies have reported that invasive species are the ‘winners’ in climate change scenarios, as they can establish populations and reproduce in climatic conditions and environments that differ from those in their native distribution range^54,55^. Researchers in diverse fields have collaborated to develop a framework that improves our understanding of invasive species and helps us evaluate, quantify, and predict their effects on native ecosystems^56^. Sophisticated algorithms and technologies have been used to collect data on invasive species^57^. Our literature search revealed that research on the sika deer has been increasing in recent years and that this species is most highly studied in Japan, which comprises their native distribution range (Figs. 1 and 2).

Climate change is a global issue that causes behavioral and/or morphological changes in organisms, large-scale shifts in species abundance and distribution, and the reorganization of ecosystems and natural resource management^58^. Furthermore, climate change facilitates the range expansion of several alien species and increases their chances of becoming invasive^59^. These features allow us to predict the current distribution and potential spread of an invasive species^17^, and such species distribution models are broadly applicable to wildlife management. In particular, habitat suitability modeling is a powerful tool for examining potential species distribution patterns. Here, we examined the impact of climate change on the potential habitat of the sika deer in South Korea. Several predictable bioclimatic variables listed in the WorldClim database and the global occurrence data of sika deer (obtained from the GBIF database) were applied to the widely used Maxent model^60,61^. As a result, the bioclimatic variables of precipitation of the driest month (bio14), mean temperature of the coldest quarter (bio11), annual mean temperature (bio1), and isothermality (bio3) were identified as major factors influencing sika deer habitats (Table 1).

The Maxent model fitness was measured based on the AUC, TSS, and Kappa values. In addition, we projected the habitat map of the sika deer using RCP 4.5 and 8.5, corresponding with the moderate and most extreme scenarios, respectively, for the predicted climatic conditions in both 2050 and 2070. The Maxent model showed a good fit in terms of the AUC (0.81), Cohen’s Kappa, and TSS values, and we benchmarked our findings based on the current distribution patterns of the species and entropy values exceeding the Maxent threshold (0.65). Using this method, we identified the northeastern and southern regions as suitable habitat zones under current climatic conditions, where deer populations already exist^16,62,63^, while the habitat loss was identified in each examined future scenario. When examined under the RCP 4.5 scenario, the habitat status of the sika deer was predicted to be better in 2070 than in 2050. Notably, RCP 4.5 is the moderate scenario in which greenhouse gas emissions peak at approximately 2040 and then decline^34,64^. This may explain the relatively higher level of habitat suitability in 2070 than in 2050. Taken together, our results indicate that climate change will influence the habitat loss of the sika deer in South Korea.

There are significant limitations to this study. We searched the Google Scholar database (English version) for publications with the keyword ‘sika deer’ in the title. However, several survey locations may have been missed by using this search strategy. We only examined species occurrence data in the GBIF database. However, several countries have developed their own local and national data portals for mammal occurrence, and several other global databases could also be searched for a more comprehensive analysis. The Maxent model used in this study is one of several habitat suitability models^65^, and multiple models should be tested to identify the optimal model for habitat suitability analysis. Furthermore, only bioclimatic variables were used to explore the impact of climatic change on sika deer habitats, and other variables with potential collinearity—such as landscape-related factors, animal behavior, snow depth, vegetation cover, and economic factors—should also be considered. Invasive species are the ‘winners’ in climate change but this study in South Korea showed the loss in habitats. Moreover, the estimation of the sika deer's potential distribution range was limited to South Korea using global data, without a comprehensive global assessment. Additionally, there is a pressing need for on-site validation, verification, and in-depth research to gain a more precise understanding of deer distribution and to formulate effective climate change mitigation strategies.

## Conclusion

Identifying the current and potential future trends in wildlife habitat distribution can help us maintain biodiversity and promote sustainable conservation strategies^66,67^. Therefore, wildlife managers and ecologists must carefully reevaluate their conservation priorities accordingly. The current study examined the global distribution of sika deer and predicted its possible future habitats within South Korea under different bioclimatic scenarios. These findings may contribute to the development of improved policies in relation to invasive species management and the climate change action plan. Thus, our results provide a reference for future policymakers and researchers and may help them design sustainable conservation strategies.

## Data Availability

Data analysed in this study are available upon reasonable request from the corresponding author (Do-Hun Lee).
